# Draft Genome Sequence of a Heterotrophic Facultative Anaerobic Thermophilic Bacterium, *Ardenticatena maritima* Strain 110S^T^

**DOI:** 10.1128/genomeA.01145-15

**Published:** 2015-10-01

**Authors:** Satoshi Kawaichi, Takashi Yoshida, Yoshihiko Sako, Ryuhei Nakamura

**Affiliations:** a Center for Sustainable Resource Science, Biofunctional Catalyst Research Team, RIKEN, Wako, Saitama, Japan; b Laboratory of Marine Microbiology, Graduate School of Agriculture, Kyoto University, Kyoto, Japan

## Abstract

*Ardenticatena maritima* strain 110S^T^ is a filamentous bacterium isolated from an iron-rich coastal hydrothermal field, and it is a unique isolate capable of dissimilatory iron or nitrate reduction among the members of the bacterial phylum *Chloroflexi*. Here, we report the draft genome sequence comprising 3,569,367 bp, containing 3,355 predicted coding sequences (CDSs).

## GENOME ANNOUNCEMENT

Members of the bacterial phylum *Chloroflexi* are ubiquitously detected from various environments, indicating their physiological versatility. Yet, a limited number of them have been isolated to date. *Ardenticatena maritima* strain 110S^T^ is isolated from an iron-rich coastal hydrothermal field and described as the first, and to date, sole isolate in the phylum to be capable of iron and nitrate reduction (1, 2). Although the 16S rRNA gene sequence of strain 110S shared low (<84%) nucleotide identity with that of other isolates, highly similar sequences were found in uncultured microbes from high-temperature metal-rich environments, such as an arsenic- and iron-rich shallow sea hydrothermal vent in Papua New Guinea (3). This knowledge implies a tight relationship between the bacterium and metals, which offers new aspects for the ecological role of the members of *Chloroflexi* (4, 5). However, our knowledge on iron and nitrate reduction by *Chloroflexi* is limited. Here, we therefore report on the sequencing and analysis of the draft genome of strain 110S.

Purified genomic DNA of strain 110S was sequenced using an Illumina MiSeq instrument and assembled with VelvetOptimiser (version 2.2.5). Among a total of 9,001,082 paired-end reads, 2,415,516 reads with Q20 value >80% were assembled into 308 contigs comprising 3,569,367 bp. Glimmer (version 2.0) predicted 3,355 coding sequences (CDSs) with minimum size of >110 bp. KEGG BlastKOALA annotated 1,439 (42%) CDSs to KO categories, including 47 tRNA-encoding genes.

In the previous report, ammonium was proposed as a possible end product of nitrate reduction (1). Still, gaseous compounds have not measured, and thus, the growth of 110S under conditions of denitrification rather than dissimilatory nitrate reduction could not be denied. In fact, the draft genome harbors a complete set of genes for the denitrification pathway (*napAB*, *nirK*, *norBC*, and *nosZ*) to enable complete denitrification from nitrate to dinitrogen. On the other hand, the assimilatory nitrite reductase gene (*nirA*), which catalyzes the reduction of nitrite to ammonium, is also present, indicating a branched pathway for assimilatory nitrate reduction and denitrification.

The microbial dissimilatory reduction of insoluble extracellular metals is well studied in Gram-negative bacteria, and outer membrane multiheme *c*-type cytochromes (MHC) are known to be responsible for the reaction. On the other hand, metal reduction mechanisms in single-membrane (monoderm) bacteria are largely unknown. Recently, Carlson et al. (6) provided evidence for cell-surface localization of cytochrome-mediated metal reductase activity in a Gram-positive bacterium, supporting MHC involvement in metal reduction by monoderm bacteria. The members of *Chloroflexi* strain Gram negative; however, they are proposed to have an atypical membrane structure, being monoderm and lacking characteristic molecules for Gram-negative cells, such as lipopolysaccharides (7–9). The absence of CDSs for the BamA protein family, which plays a crucial role in outer membrane biogenesis, and the lipopolysaccharide biosynthesis pathway in the 110S draft genome support the monoderm feature of 110S. The draft genome encodes nine multiheme proteins, including two tetraheme *c*-type cytochromes and three unidentified proteins. Further studies are ongoing to investigate the localization and involvement of these putative MHC in metal reduction by 110S.

### Nucleotide sequence accession number.

This whole-genome shotgun project has been deposited at DDBJ/EMBL/GenBank under the accession no. BBZA00000000. The version described in this paper is the first version.
